# A Comparison among Different Ways to Investigate Composite Materials with Lock-In Thermography: The Multi-Frequency Approach

**DOI:** 10.3390/ma14102525

**Published:** 2021-05-12

**Authors:** Ester D’Accardi, Davide Palumbo, Umberto Galietti

**Affiliations:** Politecnico di Bari, Department of Mechanics, Mathematics & Management, Via Orabona 4, 70125 Bari, Italy; ester.daccardi@poliba.it (E.D.); umberto.galietti@poliba.it (U.G.)

**Keywords:** lock-in thermography, multi-frequency approach, modulated frequencies, CFRP

## Abstract

The main goal of non-destructive testing is the detection of defects early enough to avoid catastrophic failure with particular interest for the inspection of aerospace structures; under this aspect, all methods for fast and reliable inspection deserve special attention. In this sense, active thermography for non-destructive testing enables contactless, fast, remote, and not expensive control of materials and structures. Furthermore, different works have confirmed the potentials of lock-in thermography as a flexible technique for its peculiarity to be performed by means of a low-cost set-up. In this work, a new approach called the multi-frequency via software approach (MFS), based on the superimposition via software of two square waves with two different main excitation frequencies, has been used to inspect a sample in carbon fiber reinforced polymers (CFRP) material with imposed defects of different materials, sizes and depths, by means of lock-in thermography. The advantages and disadvantages of the multi-frequency approach have been highlighted by comparing quantitatively the MFS with the traditional excitation methods (sine and square waves).

## 1. Introduction

Composite materials are used in many fields and engineering applications thanks to the possibility they offer to design lightweight structures with high mechanical properties. The mechanical behavior of composite structures can be affected by the presence of defects that can occur during the manufacturing process or in-service conditions. In this regard, non-destructive tests, such as thermography, can be necessary to detect and characterize defects [1].

Lock-in thermography (LT) [2,3,4,5,6,7,8,9,10,11,12,13,14,15] is among the most used active thermography techniques to inspect composite materials. It makes use of a modulated optical stimulation to heat the sample by means of a thermal wave (typically sinusoidal or square) which propagates into the material with a modulated excitation frequency. 

The capability of LT thermography and, more in general of other active thermographic techniques, to detect defects in carbon fiber reinforced polymers (CFRP) and in glass fiber reinforced polymers (GFRP) composite materials has been demonstrated in different works [13,14,15,16,17,18,19]. Several works concern, in particular, the comparison of LT with pulsed thermography (PT), in order to compare the results in terms of phase data obtained by different algorithms [3,7,10,11,12].

Maierhofer et al. [3] performed a systematic investigation of flat bottom holes (FBHs) with different diameters and remaining wall thicknesses (RWTs) in steel and CFRP and at crossed notches in steel using flash and lock-in excitation, giving interesting quantitative indications in terms of spatial resolution. 

Pickering and Almond [10] compared the signal-to-noise ratio (SNR) of flat bottom holes (FBHs) in carbon fiber reinforced polymers (CFRPs) by means of pulse and lock-in thermography techniques using matched excitation energies. The excitation energies of flash and lock-in excitation were adapted according to the energy absorbed by the specimen. With the used experimental setup and a selected frequency range of 0.01–0.1 Hz, they obtained higher SNR values for flash excitation at shallow defects and similar SNR values for both excitation methods at deeper defects. 

In the work of Ibarra-Castanedo et al. [12] in 2009, the SNRs of defects in honeycomb structures were compared using different excitation methods and techniques. 

In 2010, Montanini [7] investigated FBHs in Plexiglass; in particular, halogen lamps were used for lock-in and a ring flash with an energy of 7.2 kJ for the flash thermography. Here, some quantitative indications are given. In particular, he compared the phase images by analyzing the relative contrast of the FBHs. Furthermore, the depths of the FBHs were determined from the phase-contrast data and related blind frequency values.

In terms of processing raw thermal lock-in data, in 2015, Pitarresi [5,6] proposed a numerical off-line signal. This technique has been used for obtaining phase maps at various lock-in frequencies generated with pulse-modulated heat within one single experiment. 

Most of the works related to LT showed that, generally, this technique is performed by using a sinusoidal thermal wave with a heating source that stimulates the material at a fixed frequency value [2,3,10,11,12]. This frequency allows for investigating only a limited depths range. Furthermore, as already demonstrated in different works, it is necessary to reach the steady state conditions to have the desirable signal to noise ratio (SNR), especially in the case of a low excitation period [2,3,10]. It follows that many tests are necessary to explore thick structures and only one test requires, generally, a long time. 

To reduce the testing time, Palumbo et al. [4] proposed the use of a modulated square wave as a heat source in order to obtain from one test some information about high-order frequencies proportional to the principal one. Here, some indications about the influence of different testing and analysis parameters were given. As it was demonstrated from other works [4,5], the square wave excitation allows for obtaining significant phase data, in terms of signal to noise ratio, up to the 5th harmonic of the principal one. 

In this work, different lock-in tests have been performed to investigate a CFRP sample with imposed defects of different material, size and depth using different excitation modes. In particular, the aim is to propose a different approach to perform a lock-in test with the superimposition of two square waves (named multi-frequency approach via software (MFS)). 

In a previous conference work [14], this approach has been performed in two different ways, via hardware (controlling independently halogen lamps) and via software (composing the resulting signal via software), leading to different results discussed for a single excitation frequency. The multi-frequency approach via software appeared more versatile and applicable to any component and setup source (halogen lamps, laser) and configuration. For this reason, here we investigate the latter, showing the obtained results in terms of phase and comparing the same with the one obtained with the application of the classical approaches (sine and square wave). 

The proposed approach aims in fact to use a low-cost setup, reducing the time and duration of the inspection and data analysis. 

## 2. Theory

Generally, the LT technique is carried out by stimulating the material with a modulated sinusoidal heat source at a fixed frequency. This frequency allows to investigate the material to a given depth as reported in Equation (1):

(1) $$\varphi \left(z\right)=\frac{2\pi z}{\lambda }=\frac{z}{\mu },\;\;\mu =\sqrt{\frac{2k}{\omega \rho c_{p}}}=\sqrt{\frac{2\alpha }{\omega }}$$

where *λ* is the thermal wavelength and *μ* the thermal diffusion length, *k* is the thermal conductivity, *ρ* is the density, *c_p_* is the specific heat at constant pressure, *ω* is the modulation frequency and *α* is the thermal diffusivity. Equation (1) is only valid in the case of 1D heat transfer, i.e., for defects with lateral sizes considerably larger than the thermal diffusion length [1].

This equation indicates that higher modulation frequencies restrict the analysis in a near-surface region, while low-frequency thermal waves propagate deeper but very slowly [1]. The limits as well as the advantages of applying a classic and common approach of this type are known and already described in the introduction.

In the case of a square wave excitation, by considering the harmonics up to 5, Equation (2) allows to obtain information about amplitude and phase signal of high-order excitation frequencies [4,13,14,15]:

(2) $$T_{m}\left(t\right)=a+bt+\Delta T_{1}\sin\left(\omega t+\varphi_{1}\right)+\Delta T_{3}\sin\left(3\omega t+\varphi_{3}\right)+\ldots +\Delta T_{n}\sin\left(5\omega t+\varphi_{n}\right)$$

where *T_m_* is the mean temperature of each pixel, Δ*T_n_* and *φ_n_* are the amplitude and phase signals of a Fourier decomposition for a certain harmonic order (*n = 1,3,5, …,N*), and *a* and *b* are the constants used to model the average temperature growth of the material.

In this work, we used MultiDES system (DES S.r.l.), to acquire and analyze raw thermal lock-in data in which the equations described before are implemented and all the constants were obtained through a least-square fit method, performing an analysis pixel by pixel and considering the terms up to *n = 5* [4,13,14,15]. 

## 3. Materials and Experimental Set-Up

**Figure 1:**
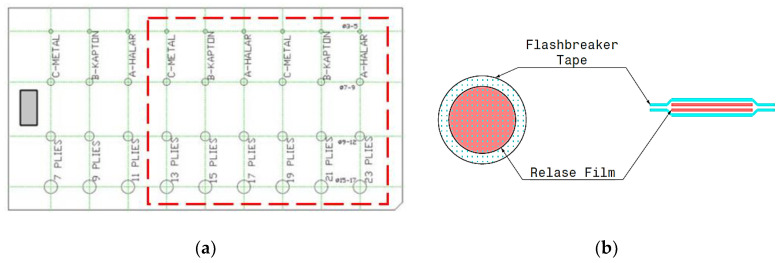
(**a**) Carbon fiber reinforced polymers (CFRP) composite sample and (**b**) scheme related to the production of the imposed defects (the part inspected is indicated with a red dotted line, total surface 250 × 500 mm^2^—inspected area 250 × 320 mm^2^).

The sample considered for the following investigations is made of CFRP composite material and it has been laminated with the use of fabric pre-peg with fiber T300 (Figure 1a). All plies (*n*. 25) are oriented at 0°/90° for a total thickness of 5 mm. The geometry of the defects is circular. The defects are produced with 2 layers of release film MR-1 RED and 2 layers of the flash breaker (Figure 1b).

Different depths in terms of interesting plies and different diameters were considered for simulating defects. Different materials, with different thermophysical properties, have been used to produce the defects (Table 1). The distance between the imposed defects is respectively 65 mm in the vertical direction and 50 mm in the horizontal direction. These distances between defects have been chosen to avoid any signal interferences between them and depend on the thermal properties of the material.

These defects were usually used for simulating delaminations in sample specimens made of CFRP or GFRP. In this case, different materials have been used to evaluate the capability in simulating the delamination for both ultrasound and thermography at the same time and also to compare this capability in respect to Teflon, which is the most common material used for thermography applications. 

**Figure 2:**
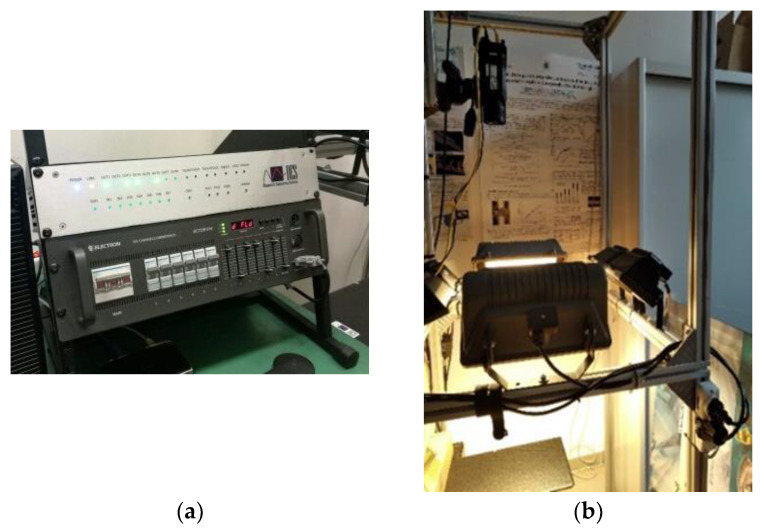
Lock-in thermography set-up: (**a**) MultiDES system and (**b**) set-up configuration.

The thermographic set-up is shown in Figure 2. Six halogen lamps with a total power of 4000 W were controlled by the MultiDES system (DES Diagnostic Engineering Solutions S.r.l.—Figure 2a,b) to stimulate the specimen. The IR camera A655sc (FLIR System, Wilsonville, OR, USA) based on a microbolometer detector was used to acquire the thermal sequences. The specimen was set as a cantilever beam configuration in order to avoid possible heat conduction effects due to supports in direct contact with its opposite side. The geometric resolution was 0.68 mm/pixel.

## 4. Experimental Campaign and Methodology

An extensive experimental campaign has been carried out for investigating different excitation modes, test and process parameters by means of a design of experiments (DOE) with full factorial design (MATLAB^®^ 2020b, Natick, MA, United States 22 Sept. 2020). In Table 2 are reported all the test parameters used for each test. In particular, a frame rate of 5 Hz has been used for all the tests and excitation periods ranging from 4.5 s to 90 s. These periods allowed for identifying defect depths up to 2.4 mm (corresponding to the left part of the specimen in Figure 1, Table 3). Three repetitions for each test were carried out. The testing plan reported in Table 2 follows a factorial plan with three factors (polynomial degree approximation of the mean temperature, frame rate and the number of cycles) with three levels (see [14]) that changed during some tests and analyses, as shown in Table 2. In this work, the attention was only focused on the different excitation methods (as indicated in Table 4), leaving to further works the analysis of the other test parameters.

The three different modes of excitation, used for the tests, correspond to sinusoidal, square and multi-frequency waves. As already specified, the last one has been obtained by superimposing, via software, two square wave signals at different excitation periods (MFS) and then allowing to obtain the phase data related to the higher odd harmonics (Equation (2)). The choice of the main periods for MFS tests derives from the previous studies [14], in which has been demonstrated that the period of 45 s allows for detecting a great number of indications. In this regard, the two tests with the MFS approach have been carried out by superimposing periods of 22.5 s and 45 s (22.5–45 s test) and 45 s and 90 s (45–90 s test) and modulating the lamps from the 0% to the 100% of the total power (0–4000 W). Considering Equation (2), the phase data for each period have been assessed up to the fifth harmonic, so for each MFS test, a total of six phase maps have been considered (three for each main excitation period). The tests with the classical approach have been carried out by modulating the power of the lamps between the 0% and 50% in order to have the same provided power for each modulated period.

**Figure 3:**
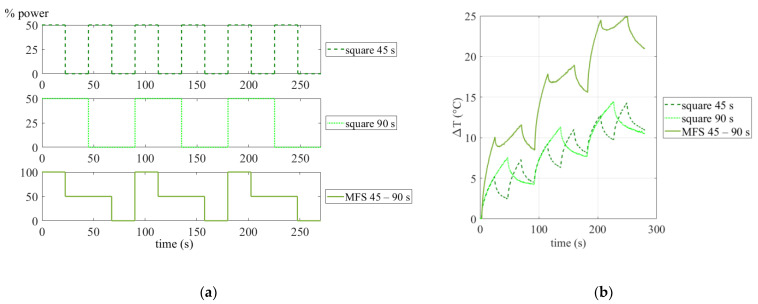
(**a**) Scheme of the multi-frequency software MFS (multi-frequency software) test 45–90 s and (**b**) the temperature signals starting from the superimposition of two different square waves with periods 45 s and 90 s.

The schematic explanation of the multi-frequency experiment via software is reported in Figure 3a, for the couples of square waves excitation periods 45–90 s. As shown, the multi-frequency via software is the result of a superimposition of two different square waves, by controlling the lamps (on-off) as shown in the final graph.

Instead, in Figure 3b, the temperature signal is reported by analyzing a 3 × 3 matrix in a sound area of the sample, comparing the obtained MFS signal (45–90 s) with the starting square signals (single periods 45 s and 90 s). Clearly, the use of two different periods of excitation during the same test determines higher temperatures than the single period, as shown in Figure 3b, even though the maximum power used for each period is the same, when the same are considered individually.

The comparison regarded the phase results in terms of the defect detection and the reached normalized phase contrast (CNR). 

**Figure 4:**
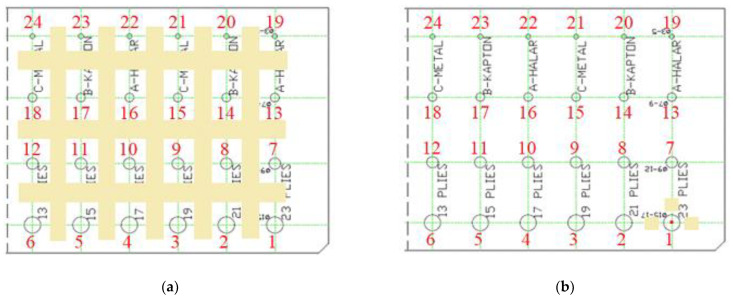
Choice for the sound area: (**a**) First case, transition from phase signal to phase contrast maps; (**b**) Second case, analysis defect by defect and calculation of the normalized contrast; the numbers used to indicate each defect is also reported.

The phase contrast maps were obtained subtracting the average of the sound area, calculated as the sum of all the areas surrounding every single defect—i.e., Figure 4a (for a total of about 20,000 pixels). In this way, it is possible to compare all the results and maps keeping the same scale. Finally, a comparison defect by defect (matrix 3 × 3 as mean value for each defect) has been performed, considering for each individual defect only its sound area (about 1000 pixels around the considered defect as shown in Figure 4b), avoiding the edge effects.

## 5. Results

### 5.1. Preliminary Considerations

**Figure 5:**
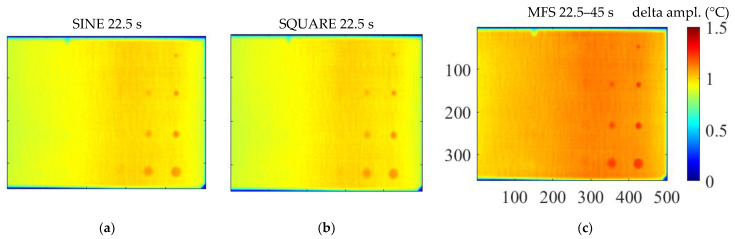
Delta amplitude results considering 22.5 s as period of excitation: (**a**) Sine; (**b**) Square; (**c**) MFS.

As already specified and highlighted in Figure 4, only a part of the specimen was considered for comparisons and in this way, an attempt was made to excite only the part of the specimen considered, as uniformly as possible. Figure 5 reports three amplitude images (adopting the scheme in Figure 4a for the definition of the sound area) related to the three different waveforms and considering as main excitation period 22.5 s.

Amplitude images allow for evaluating the heating distribution on the specimen due to the adopted set-up. In this regard, as expected, the central area of the inspected part of the specimen is characterized by a higher temperature than the edges and the part of the specimen not interested by heating. These Δ*T* differences within the specimen can become significant for the higher periods in which higher temperatures are reached and can induce the in-plane heat diffusion with consequent phase signal variations. The effect is more significant in the case of the multi-frequency approach where two periods of excitation are combined and then the specimen reaches higher temperatures than the other methods based on the single periods. This behavior is evident in the results reported in terms of the delta amplitude and the delta phase signal. Consequently, we expect that the phase data obtained with the MFS approach will be affected by a high level of noise than the other approaches.

**Figure 6:**
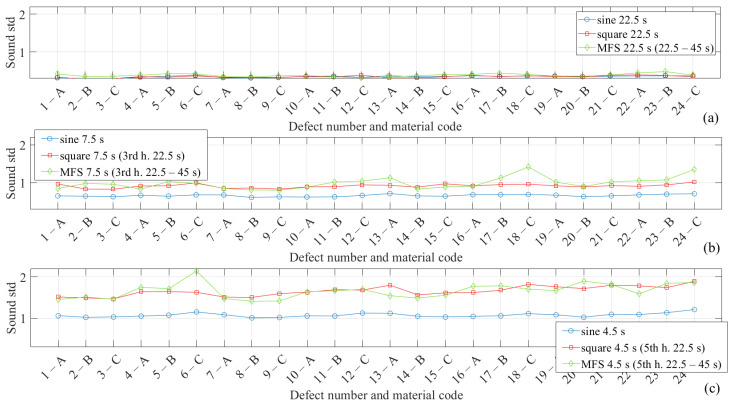
Sound standard deviation considering different areas for each defect, specifying numbers and material code as reported in Figure 4: (**a**) 22.5 s—1st harmonic; (**b**) 7.5 s—3rd harmonic; (**c**) 4.5 s—5th harmonic.

In this regard, a first comparison among the three different modes of excitations is depicted in Figure 6 in which is reported the standard deviation values (std) of the sound area related to each defect, as shown in Figure 4b. In particular, Figure 6a–c concerns the excitation periods and therefore the related harmonics, as specified in the case of the square or multi-frequency approach. Differences can only be appreciated for the higher-order harmonics, between sine and square and sine and MFS, and in correspondence with the defects at the left edge of the specimen. This result confirms that the distance between the imposed defects is sufficient to avoid interferences between them.

### 5.2. Phase Results

**Figure 7:**
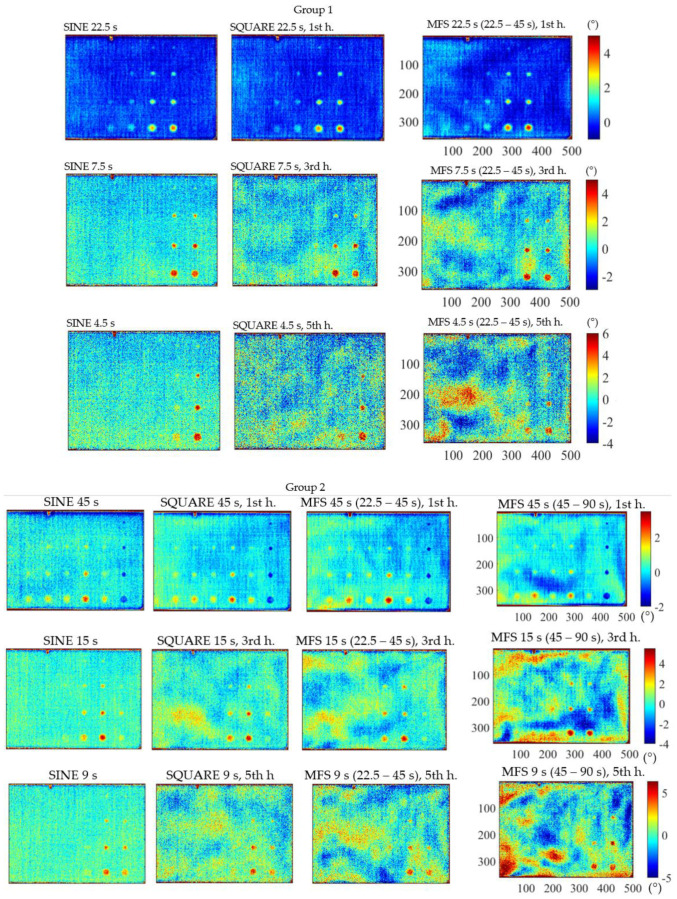

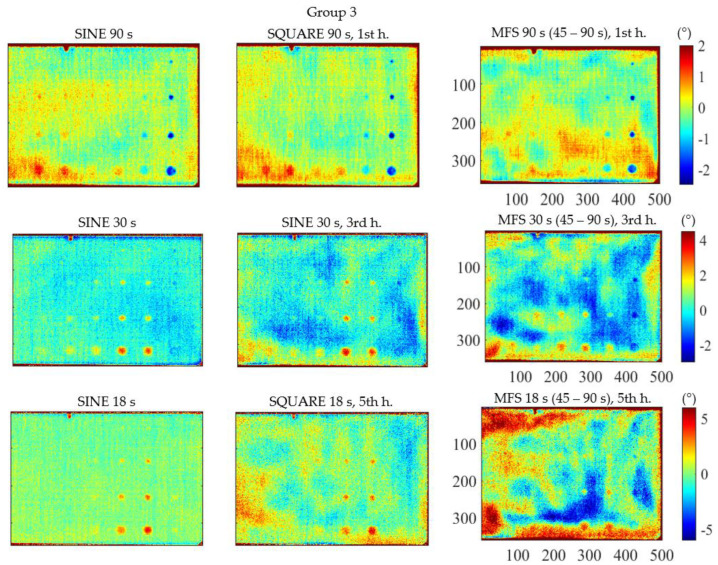
Results for the three different waveforms, sine, square and MFS and for each period. The test parameters for each analysis are reported in Table 4: Group 1—22.5, 7.5, 4.5 s; Group 2—45, 15, 9 s; Group 3—90, 30, 18 s.

The phase results reported in Figure 7 as delta phase maps were arranged within a matrix scheme in which the column represents waveforms, and the row represents the excitation periods. A total of 30 delta phase images were obtained. For a direct qualitative comparison, once the excitation period was fixed, the scale has been fixed independently of the analyzed waveform. To compare all the phase data, the normalized contrast has been used and assessed defect by defect, with the aim to obtain indications about the detectability of defects. The test parameters used for the analysis are reported in Table 4. Qualitatively, the MFS approach results competitive if compared to the traditional method if the first harmonic is considered. On the other hand, as expected, the phase data related to the higher harmonics, are affected by a significant noise, above all for the fifth harmonic.

## 6. Quantitative Analysis and Discussion

### 6.1. Advantages and Disadvantages of the MFS Approach

**Figure 8:**
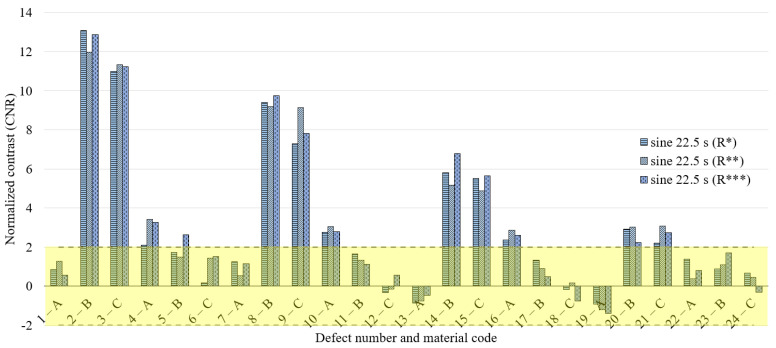
Results for the excitation period equal to 22.5 s in the case of a sine wave; 3 replications and normalized phase contrast value for each defect.

**Figure 9:**
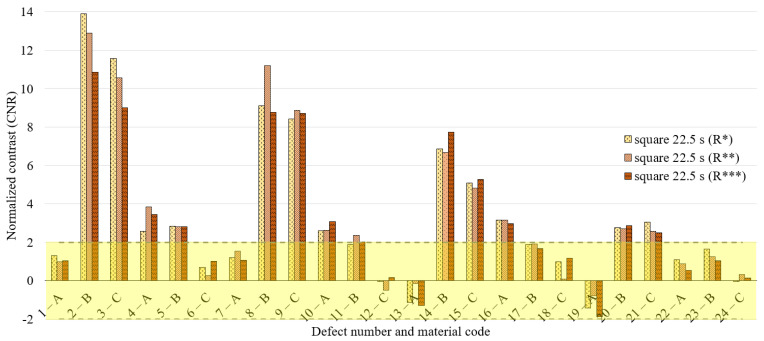
Results for the excitation period equal to 22.5 s in the case of a square wave; 3 replications and normalized phase contrast value for each defect.

**Figure 10:**
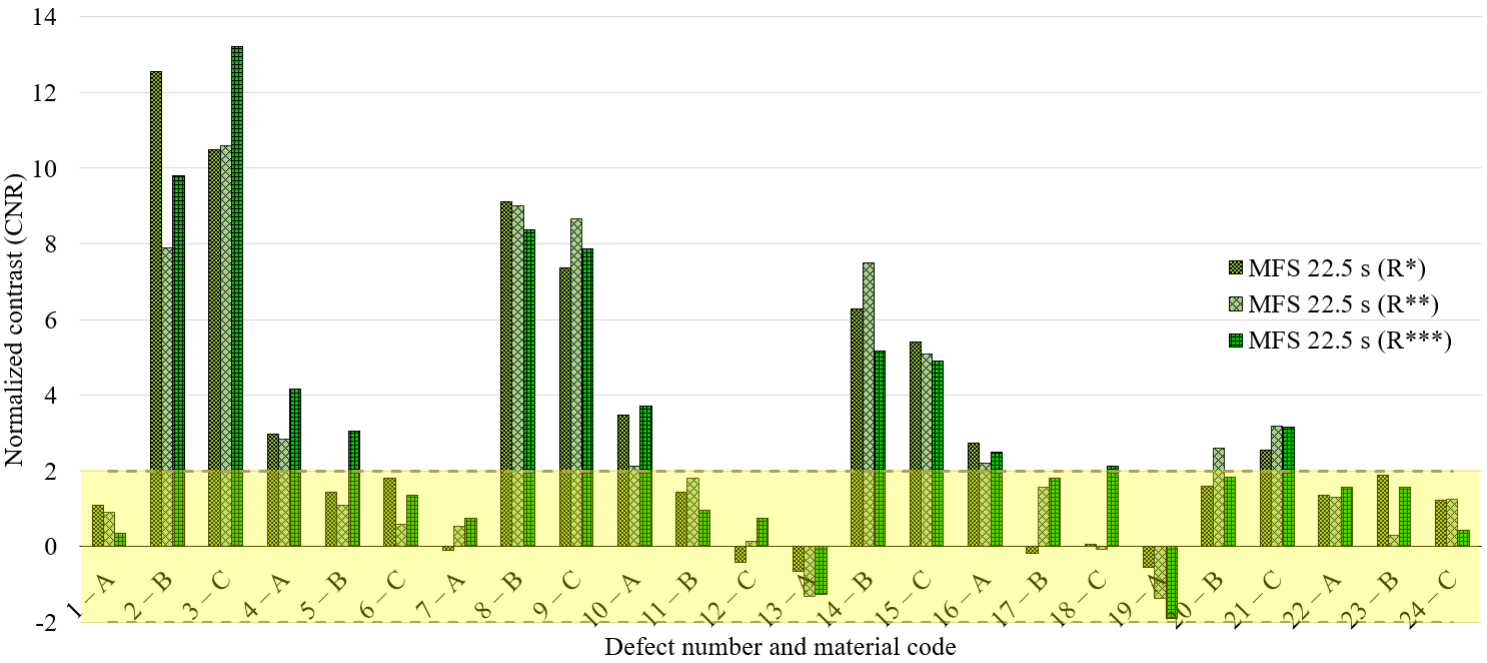
Results for the excitation period equal to 22.5 s in the case of MFS wave 22.5–45 s; 3 replications and normalized phase contrast value for each defect.

To discuss the obtained results, we decide to refer to the normalized contrast, defect by defect, identifying the defect and sound area as described previously and reported in Figure 4b. Figure 8, Figure 9 and Figure 10 concern the obtained results for the main period of 22.5 s, analyzing all the defects and the replications (replication 1—R*, replication 2—R** and replication 3—R***) for each waveform (Figure 8—sine, Figure 9—square, Figure 10—MFS 22.5—45 s). Moreover, for each defect, three test replications executed with equal conditions have been considered and analyzed in the same way and with the same reference areas. A defect has been considered detectable if its contrast exceeds twice the value of the standard deviation, as reported in Figure 8, Figure 9 and Figure 10 with a transparent yellow area.

The differences among replications are sometimes significant especially in the case of the MFS approach. These differences concern not the mean values, but the standard deviation ones, which can be affected by small variations due, for example, to surface conditions (despite the waiting time between one test and the next) and afterglow effects of the lamps that occur after many tests. 

In Table 5, the previous results are summarized, defect by defect, setting equal to 1 the condition for which the defect is detected, CNR ≥ 2, and with 0, the opposed event. The last column for each waveform is the result of the sum of the three replications, and therefore represents the probability to detect a defect when the same test is repeated and analyzed under the same conditions three times. The total number of detected defects are indicated in the last row. In this case, it has been sufficient to consider one replication with a positive result as a defect as a detected one.

**Figure 11:**
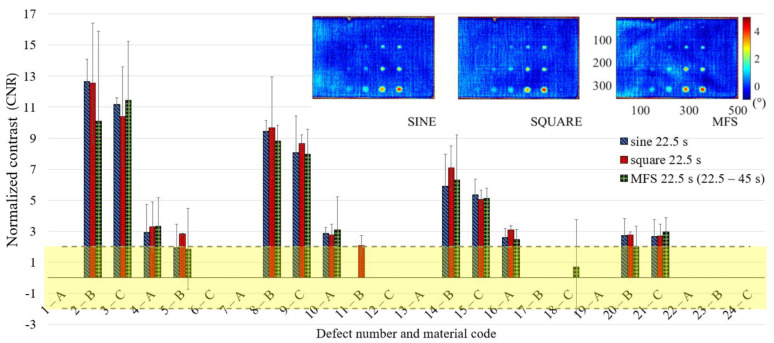
Results in terms of CNR (normalized phase contrast) and related confidence bounds reporting only the defects with a CNR ≥ 2 (only one replication with a positive result in this sense is sufficient); a comparison among sine, square and MFS for the period equal to 22.5 s.

**Figure 12:**
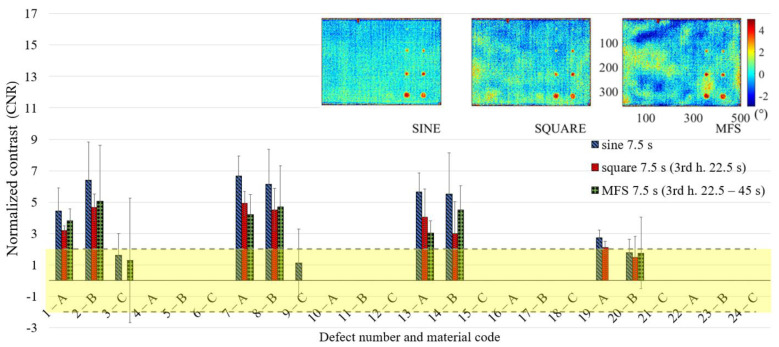
Results in terms of CNR and related confidence bounds reporting only the defects with a CNR ≥ 2 (only one replication with a positive result in this sense is sufficient); a comparison among sine, square (3rd harmonic) and MFS (3rd harmonic) for the period equal to 7.5 s.

**Figure 13:**
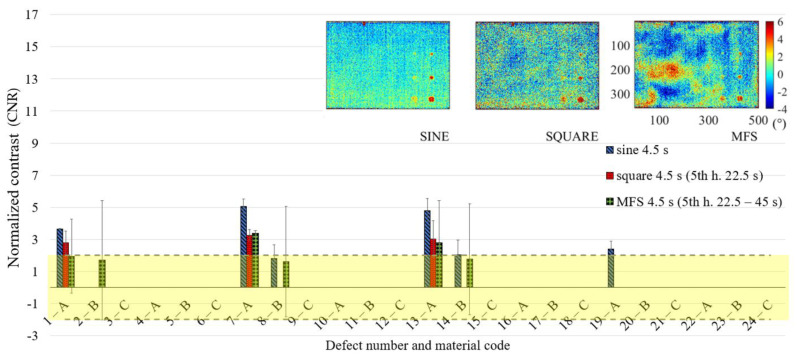
Results in terms of CNR and related confidence bounds reporting only the defects with a CNR ≥ 2 (only one replication with a positive result in this sense is sufficient); a comparison among sine, square (5th harmonic) and MFS (5th harmonic) for the period equal to 4.5 s.

In Figure 11 are reported only the defects with the CNR ≥ 2, with the confidence bounds that indicate the test uncertainty, assuming for their definition, a Student-t distribution of the analyzed data set and a confidence level of 95%. In this way, due to the limited number of replications, each defect is characterized by large confidence bounds with a consequent high probability to be included as a detected defect with 95% of confidence. This effect is more evident for high order harmonics, as shown in Figure 12 and Figure 13, where the obtained results related to the periods of 7.5 and 4.5 s are reported. However, Figure 11, Figure 12 and Figure 13 allow both to obtain useful information about the defect detectability and to evaluate the performance of the new proposed approach in comparison with the well-established ones. 

Similar considerations come from the analyses of the other excitation periods that are summarized in Table 6, where it is possible to find the overall results for each analyzed defect. The results are always organized in groups, considering the main period and the related higher order harmonics on the same line. The columns concerning the results for a sine wave are always intended as analyses of main periods and then as single separated tests.

The last row for each group shows the number of detected defects for a given waveform and excitation period. As already specified in Table 5, it is sufficient to have a positive result for a replication (P = 1/3) to consider the related defect in the total sum. 

Summarizing, all the results show the good ability of the MFS approach in terms of defect detection and the CNR if it is compared to well-established approaches used for LT. As expected, the quality of phase data decreases passing through the first to the third and fifth harmonic; these latter are characterized by a higher level of noise. 

### 6.2. Comparing the Two Different Pairs of Main Excitation Periods 22.5–45 s, 45–90 s in the MFS Approach 

As already said, the choice of the main periods of excitation for an MFS-type approach depends on the expected defect depth.

**Figure 14:**
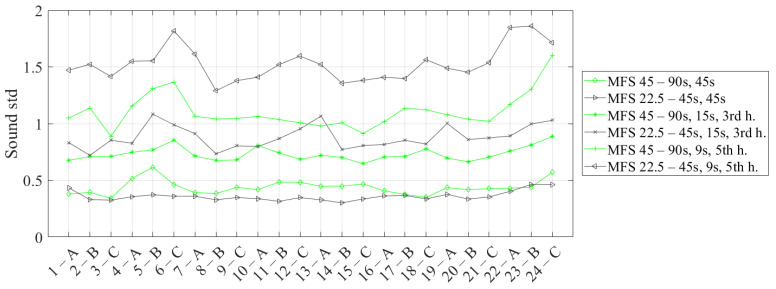
Standard deviation trends considering a different sound area for each defect and the main and, subsequently, harmonics in the case of the MFS test (22.5–45 s vs. 45–90 s).

Figure 14 shows the standard deviation (std) trends in both cases, comparing the standard deviation values of the sound for each defect and for the main and, subsequently, harmonics. Here, the mean values are reported considering the three replications carried out for each test. The results are comparable for the first harmonic, with values that seem slightly lower in the case of the MFS test 22.5–45 s. In the other cases, the test MFS at 45–90 s presents always lower standard deviation values for each defect. As already said, these results are probably due to the different average temperature reached in the two MFS tests. This interesting effect that affects the phase signal will be the subject of future studies.

**Figure 15:**
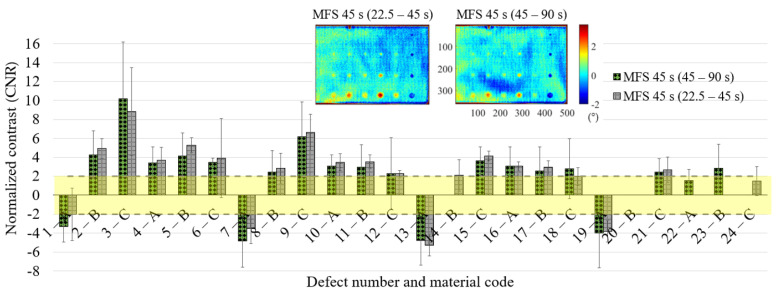
Results in terms of CNR and related confidence bounds reporting only the defects with a CNR ≥ 2; comparison between the MFS tests (22.5–45 s vs. 45–90 s) for the main period of 45 s.

**Figure 16:**
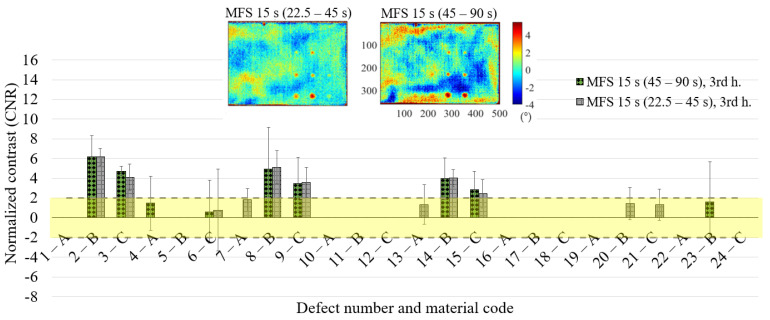
Results in terms of CNR and related confidence bounds reporting only the defects with a CNR ≥ 2; comparison between the MFS tests (22.5–45 s vs. 45–90 s) for the 3rd harmonic 15 s.

**Figure 17:**
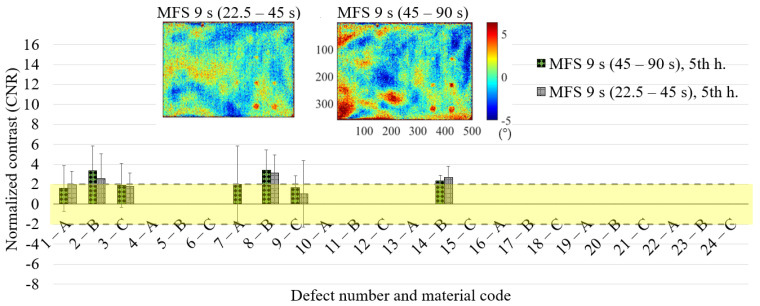
Results in terms of CNR and related confidence bounds reporting only the defects with a CNR ≥ 2 (only one replication with a positive result in this sense is sufficient); a comparison between the MFS tests (22.5–45 s vs. 45–90 s) for the 5th harmonic 9 s.

The results in terms of CNR for each defect (Figure 15, Figure 16 and Figure 17) are substantially comparable in both cases, with a few more false positives in the case of the 22.5–45 s MFS test due to the higher noise level already mentioned before (see Figure 16—6C, 7A, 13A, 20B, 21C). These are the defects with a mean CNR value < 2, but that in one replication shows a CNR value > 2. The comparison is completed in the previous Table 6, where the results are reported for each defect for the two MFS tests. Above all, for deeper defects, such as 6C, 12C and 18C, the MFS 45–90 s test provides higher CNR values.

### 6.3. Comparing the Obtained Results in Terms of Excitation Period and Waveforms Defect by Defect

**Figure 18:**
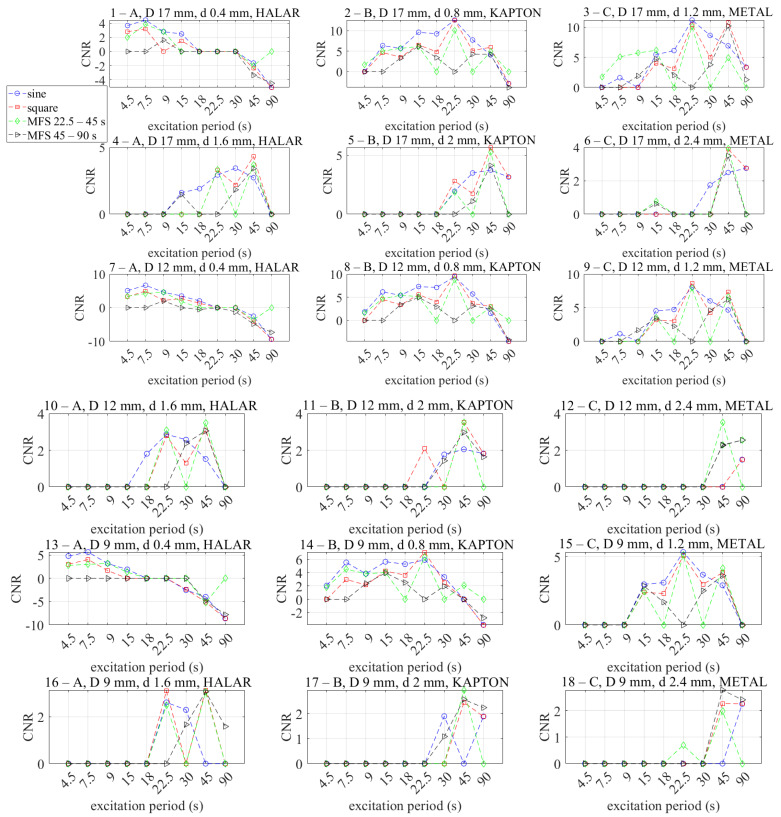

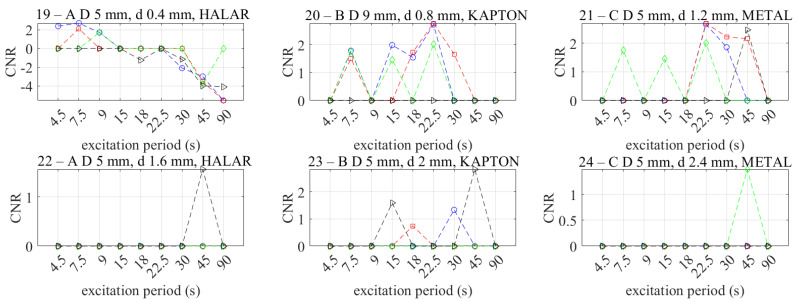
Comparing the results in terms of CNR considering all the excitation periods and all the defects.

Figure 18 summarizes the previous results and provides an overview in terms of waveforms and excitation periods for each defect. 

First of all, it is necessary to underline the particular behavior of the defects indicated as 1-A, 6-A, 13-A and 19-A, which are the shallower (0.4 mm). For these defects, independently from the examinated waveforms, as expected, the higher positive CNR values correspond to lower excitation periods, then follows a “blind zone” for the periods of 18, 22.5 and 30 s where the CNR values are lower than the chosen threshold value and change in sign. 

For some defects, for example, the defect indicated as 6-C, the CNR values for some periods are higher for the MFS approach than the sine or square wave ones. As already explained above, this behaviour can be explained by considering the average temperature effect.

Finally, the last 3 graphs compared with the related phase maps in Figure 7 (defects 22-A, 23-B, 24-C) are to be understood as false positives, with the lower mean CNR values (more or less <2), but with a high confidence band. 

## 7. Conclusions and Future Works

In this work, advantages and limits of the multi-frequency via software test (MFS) were shown and discussed in comparison with classical approaches based on the sine and square waves excitation. The MFS approach has been proposed with the aim to reduce the time of testing and analysis associated with lock-in tests, which generally involve several tests to inspect thick structures. 

A specimen made of CFRP material with different imposed defects with different depth, size and materials has been investigated, performing several tests with all the approaches and different excitation periods in the same conditions, adopting halogen lamps and a microbolometer detector (FLIR A655sc). The results have been compared in a quantitative way considering the contrast to noise ratio value (CNR) and the number of detected defects. 

The main results can be here summarized:

- The MFS approach allows for investigating six phase maps from a single test with respect to the traditional approaches, sine (one phase map) and square (three phase maps) waves. In turn, it results in a reduced testing time for screening and analysis of several components or large structures.
- Considering the phase data of the main (first) harmonic, the MFS approach provides comparable results in terms of CNR and the number of detected defects in comparison with the traditional approaches.
- CNR values obtained with the MFS decrease as the order of harmonic increases due to a high level of noise of the phase data. Differences with respect to the sine and square approaches begin to be significant, above all, for the fifth harmonic. In this regard, a reduced capability in detecting the smaller and deeper defects has been observed.

Finally, it is important to highlight that, although the different approaches were compared using the same external excitation power, different temperature variations and a different increase of the mean temperature were observed in each test, MFS, sine and square wave. These differences were due both to the different shapes of the excitation waves and to the superimposition of two different periods for the MFS approach. This interesting point that can affect the quality of phase data will be investigated better in future works evaluating the absorbed energy level for each excitation period related to each waveform. 

## Figures and Tables

**Table 1:** Material, defect depths and diameters.

Material	Defect Depth (Starting from the Inspected Side)			Defect Diameter (Starting from the First Row in Figure 1)	
				Release Film (mm)	Flashbreaker Tape (mm)
A–HALAR	0.4 mm 23 Pl.	1.6 mm 17 Pl.	2.8 mm 11 Pl.	3	5
B–KAPTON	0.8 mm 21 Pl.	2 mm 15 Pl.	3.2 mm 9 Pl.	7	9
C–METAL	1.2 mm 19 Pl.	2.4 mm 13 Pl.	3.6 mm 7 Pl.	9	12
				15	17

**Table 2:** Experimental campaign.

Waveforms	Excitation Periods (s)	Acquisition Duration (s)	Frame Rate (Hz)	Number of Cycles	% Power	Excitation Periods (s—after the Test, Available for the Analyses)
Multi-frequency software (MFS)	22.5–45	135	5 Hz	6–3	Figure 3a	4.5, 7.5, 9, 15, 22.5, 45
	45–90	270				9, 15, 18, 30, 45, 90
Sine	4.5	135	5 Hz	30	50	4.5
	7.5	135		18		7.5
	9	270		30		9
	15	270		18		15
	18	270		15		18
	22.5	135		6		22.5
	30	270		9		30
	45	270		6		45
	90	270		3		90
Square	22.5	135	5 Hz	6	50	4.5, 7.5, 22.5
	45	270		6		9, 15, 45
	90	270		3		18, 30, 90

**Table 3:** Excitation period for the different defect depths (Equation (1)).

**Thermal Diffusivity CFRP α (mm^2^/s)**	0.42								
**Depth (mm)**	3.6	3.2	2.8	2.4	2.0	1.6	1.2	0.8	0.4
**Period (s)**	96.9	76.6	58.6	43.1	29.9	19.1	10.8	4.8	1.2

**Table 4:** Testing and analysis plan chosen to compare the excitation modes.

Waveforms	Excitation Periods (s)	Frame Rate (Hz)	Number of Cycles	% Power	Harmonic Order
Multi-frequency software (MFS) Sine Square	22.5	5 Hz	3	Figure 3 50% for each modulation frequency (period of excitation)	1st
	45				1st
	90				1st
	4.5				5th (22.5 s)
	7.5				3rd (22.5 s)
	9				5th (45 s)
	15				3rd (45 s)
	18				5th (90 s)
	30				3rd (90 s)

**Table 5:** Table that summarizes the previous results in the case of a period equal to 22.5 s for three replications.

	Sine 22.5 s				Square 22.5 s				Multi-Frequency 22.5 s (22.5–45 s)			
Defect	R*	R**	R***	Probability	R*	R**	R***	Probability	R*	R**	R***	Probability
1-A	0	0	0	0	0	0	0	0	0	0	0	0
2-B	1	1	1	1	1	1	1	1	1	1	1	1
3-C	1	1	1	1	1	1	1	1	1	1	1	1
4-A	1	1	1	1	1	1	1	1	1	1	1	1
5-B	0	0	1	1/3	1	1	1	1	0	0	1	1/3
6-C	0	0	0	0	0	0	0	0	0	0	0	0
7-A	0	0	0	0	0	0	0	0	0	0	0	0
8-B	1	1	1	1	1	1	1	1	1	1	1	1
9-C	1	1	1	1	1	1	1	1	1	1	1	1
10-A	1	1	1	1	1	1	1	1	1	1	1	1
11-B	0	0	0	0	0	1	1	2/3	0	0	0	0
12-C	0	0	0	0	0	0	0	0	0	0	0	0
13-A	0	0	0	0	0	0	0	0	0	0	0	0
14-B	1	1	1	1	1	1	1	1	1	1	1	1
15-C	1	1	1	1	1	1	1	1	1	1	1	1
16-A	1	1	1	1	1	1	1	1	1	1	1	1
17-B	0	0	0	0	0	0	0	0	0	0	0	0
18-C	0	0	0	0	0	0	0	0	0	0	1	1/3
19-A	0	0	0	0	0	0	0	0	0	0	0	0
20-B	1	1	1	1	1	1	1	1	0	1	0	1/3
21-C	1	1	1	1	1	1	1	1	1	1	1	1
22-A	0	0	0	0	0	0	0	0	0	0	0	0
23-B	0	0	0	0	0	0	0	0	0	0	0	0
24-C	0	0	0	0	0	0	0	0	0	0	0	0
NUM. TOT.	11	11	12	12	12	13	13	13	10	11	12	12

**Table 6:** Comparing all the achieved results in terms of CNR and probability of detection for each defect and excitation period.

Defect Identification and Dimensions (mm)				Excitation Period (s)																	
	**D.** **EX.**	**d.**	**Material**	**Sine 4.5 s**		**Square 4.5 s (22.5 s)—5th**		***MFS 4.5 s (22.5–45 s)—*** **5th**		**Sine 7.5 s**		**Square 7.5 s (22.5 s)—3rd**		***MFS 7.5 s (22.5–45 s)*** **—3rd**		**Sine 22.5 s**		**Square 22.5 s**		***MFS 22.5 s*** ***(22.5–45 s)***	
				**P**	**CNR (m. v.)**	**P**	**CNR (m. v.)**	***P***	***CNR (m. v.)***	**P**	**CNR (m. v.)**	**P**	**CNR (m. v.)**	***P***	***CNR*** ***(m. v.)***	**P**	**CNR (m. v.)**	**P**	**CNR** **(m. v.)**	***P***	***CNR*** ***(m. v.)***
1-A	17	0.4	HALAR	1	3.66	1	2.80	*1/3*	*1.96*	1	4.45	1	3.18	*1*	*3.82*						
2-B	17	0.8	KAPTON					*1/3*	*1.71*	1	6.39	1	4.66	*1*	*5.05*	1	12.64	1	12.55	*1*	*10.09*
3-C	17	1.2	METAL							1/3	1.61			*1/3*	*1.30*	1	11.18	1	10.38	*1*	*11.43*
4-A	17	1.6	HALAR													1	2.92	1	3.29	*1*	*3.33*
5-B	17	2	KAPTON													1/3	1.95	1	2.82	*1/3*	*1.86*
6-C	17	2.4	METAL																		
7-A	12	0.4	HALAR	1	5.07	1	3.27	*1*	*3.37*	1	6.66	1	4.92	*1*	*4.21*						
8-B	12	0.8	KAPTON	2/3	1.81			*1/3*	*1.61*	1	6.14	1	4.51	*1*	*4.69*	1	9.45	1	9.69	*1*	*8.83*
9-C	12	1.2	METAL							1/3	1.14					1	8.08	1	8.67	*1*	*7.97*
10-A	12	1.6	HALAR													1	2.86	1	2.77	*1*	*3.10*
11-B	12	2	KAPTON															2/3	2.09		
12-C	12	2.4	METAL																		
13-A	9	0.4	HALAR	1	4.81	1	3.02	*2/3*	*2.79*	1	5.65	1	4.05	*1*	*3.01*						
14-B	9	0.8	KAPTON	2/3	2.05			*1/3*	*1.77*	1	5.52	1	2.98	*1*	*4.51*	1	5.91	1	7.09	*1*	*6.31*
15-C	9	1.2	METAL													1	5.34	1	5.07	*1*	*5.13*
16-A	9	1.6	HALAR													1	2.61	1	3.10	*1*	*2.48*
17-B	9	2	KAPTON																		
18-C	9	2.4	METAL																	*1/3*	*0.7*
19-A	5	0.4	HALAR	1	2.40					1	2.72	2/3	2.12								
20-B	5	0.8	KAPTON							1/3	1.79	1/3	1.50	*1/3*	*1.76*	1	2.72	1	2.77	*1/3*	*2.01*
21-C	5	1.2	METAL													1	2.67	1	2.71	*1*	*2.96*
22-A	5	1.6	HALAR																		
23-B	5	2	KAPTON																		
24-C	5	2.4	METAL																		
NUM. TOT.				6		3		*6*		10		7		*8*		12		13		*12*	
	**D.** **EX.**	**d.**	**MATERIAL**	**SINE 9 s**		**SQUARE 9 s (45 s)—5th**		***MFS 9 s (22.5–45 s)*** **—5th**		**SINE 15 s**		**SQUARE 15 s (45 s)—3rd**		***MFS 15 s (22.5–45 s)*** **—3rd**		**SINE 45 s**		**SQUARE 45 s**		***MFS 45 s*** ***(22.5–45 s)***	
				**P**	**CNR (m. v.)**	**P**	**CNR (m. v.)**	***P***	***CNR*** ***(m. v.)***	**P**	**CNR** **(m. v.)**	**P**	**CNR** **(m. v.)**	***P***	***CNR*** ***(m. v.)***	**P**	**CNR (m. v.)**	**P**	**CNR (m. v.)**	***P***	***CNR*** ***(m. v.)***
1-A	17	0.4	HALAR	1	2.81			*2/3*	*1.92*	1	2.47	1/3	1.51			1/3	−1.63	2/3	−2.35	*2/3*	*−2.01*
2-B	17	0.8	KAPTON	1	5.71	1	3.57	*2/3*	*2.55*	1	9.64	1	6.55	*1*	*6.15*	1	4.10	1	5.96	*1*	*4.92*
3-C	17	1.2	METAL					*2/3*	*1.79*	1	5.44	1	4.01	*1*	*4.11*	1	6.90	1	10.77	*1*	*8.85*
4-A	17	1.6	HALAR							1/3	1.61					1	2.73	1	4.32	*1*	*3.69*
5-B	17	2	KAPTON													1	3.75	1	5.69	*1*	*5.26*
6-C	17	2.4	METAL											*1/3*	*0.77*	1	2.49	1	3.88	*1*	*3.93*
7-A	12	0.4	HALAR	1	4.61	2/3	2.16			1	3.47	1	2.72	*1/3*	*1.82*	2/3	−2.53	1	−4.17	*1*	*−3.56*
8-B	12	0.8	KAPTON	1	5.41	1	3.33	*1*	*3.16*	1	7.29	1	5.55	*1*	*5.13*	1/3	1.54	1	3.00	*1*	*2.83*
9-C	12	1.2	METAL					*1/3*	*1.06*	1	4.54	1	3.16	*1*	*3.58*	1	4.66	1	7.34	*1*	*6.64*
10-A	12	1.6	HALAR													1/3	1.52	1	3.10	*1*	*3.50*
11-B	12	2	KAPTON													2/3	2.05	1	3.52	*1*	*3.53*
12-C	12	2.4	METAL																	*1*	*2.26*
13-A	9	0.4	HALAR	1	3.18	1/3	1.66			1/3	1.92			*1/3*	*1.34*	1	−3.97	1	−5.07	*1*	*−5.26*
14-B	9	0.8	KAPTON	1	3.83	2/3	2.14	*1*	*2.68*	1	5.63	1	4.23	*1*	*4.02*					*1/3*	*2.10*
15-C	9	1.2	METAL							1	2.98	2/3	2.39	*2/3*	*2.43*	1	2.90	1	3.83	*1*	*4.16*
16-A	9	1.6	HALAR															1	3.13	*1*	*3.05*
17-B	9	2	KAPTON															2/3	2.45	*1*	*2.94*
18-C	9	2.4	METAL															2/3	2.27	*1/3*	*1.99*
19-A	5	0.4	HALAR	1/3	1.71											1	−3.00	1	−3.53	*1*	*−3.80*
20-B	5	0.8	KAPTON							2/3	1.99			*1/3*	*1.45*						
21-C	5	1.2	METAL											*1/3*	*1.33*			2/3	2.16	*1*	*2.67*
22-A	5	1.6	HALAR																		
23-B	5	2	KAPTON																		
24-C	5	2.4	METAL																	*1/3*	*1.49*
NUM. TOT.				7		5		*6*		11		8		*11*		14		18		*21*	
	**D.** **EX.**	**d.**	**MATERIAL**	**SINE 9 s**		**SQUARE 9 s (45 s)—5th**		***MFS 9 s*** ***(45–90 s)*** **—5th**		**SINE 15 s**		**SQUARE 15 s (45 s)—3rd**		***MFS 15*** ***(45–90 s)*** **—3rd**		**SINE 45 s**		**SQUARE 45 s**		***MFS 45 s*** ***(45–90 s)***	
				**P**	**CNR (m. v.)**	**P**	**CNR** **(m. v.)**	***P***	***CNR*** ***(m. v.)***	**P**	**CNR** **(m. v.)**	**P**	**CNR** **(m. v.)**	***P***	***CNR*** ***(m. v.)***	**P**	**CNR** **(m. v.)**	**P**	**CNR** **(m. v.)**	***P***	***CNR*** ***(m. v.)***
1-A	17	0.4	HALAR	1	2.81			*1/3*	*1.59*	1	2.47	1/3	1.51			1/3	−1.63	2/3	−2.35	*1*	*−3.33*
2-B	17	0.8	KAPTON	1	5.71	1	3.57	*1*	*3.36*	1	9.64	1	6.55	*1*	*6.18*	1	4.10	1	5.96	*1*	*4.29*
3-C	17	1.2	METAL					*1/3*	*1.89*	1	5.44	1	4.01	*1*	*4.72*	1	6.90	1	10.77	*1*	*10.20*
4-A	17	1.6	HALAR							1/3	1.61			*1/3*	*1.47*	1	2.73	1	4.32	*1*	*3.41*
5-B	17	2	KAPTON													1	3.75	1	5.69	*1*	*4.13*
6-C	17	2.4	METAL											*1/3*	*0.61*	1	2.49	1	3.88	*1*	*3.49*
7-A	12	0.4	HALAR	1	4.61	2/3	2.16	*2/3*	*2.02*	1	3.47	1	2.72			2/3	−2.53	1	−4.17	*1*	*−4.81*
8-B	12	0.8	KAPTON	1	5.41	1	3.33	*1*	*3.39*	1	7.29	1	5.55	*1*	*4.95*	1/3	1.54	1	3.00	*2/3*	*2.44*
9-C	12	1.2	METAL					*1/3*	*1.67*	1	4.54	1	3.16	*1*	*3.45*	1	4.66	1	7.34	*1*	*6.20*
10-A	12	1.6	HALAR													1/3	1.52	1	3.10	*1*	*3.06*
11-B	12	2	KAPTON													2/3	2.05	1	3.52	*1*	*2.97*
12-C	12	2.4	METAL																	*2/3*	*2.28*
13-A	9	0.4	HALAR	1	3.18	1/3	1.66			1/3	1.92					1	−3.97	1	−5.07	*1*	*−4.79*
14-B	9	0.8	KAPTON	1	3.83	2/3	2.14	*1*	*2.36*	1	5.63	1	4.23	*1*	*3.95*						
15-C	9	1.2	METAL							1	2.98	2/3	2.39	*1*	*2.85*	1	2.90	1	3.83	*1*	*3.62*
16-A	9	1.6	HALAR															1	3.13	*1*	*3.06*
17-B	9	2	KAPTON															2/3	2.45	*2/3*	*2.57*
18-C	9	2.4	METAL															2/3	2.27	*2/3*	*2.78*
19-A	5	0.4	HALAR	1/3	1.71											1	−3.00	1	−3.53	*1*	*−3.99*
20-B	5	0.8	KAPTON							2/3	1.99										
21-C	5	1.2	METAL															2/3	2.16	*2/3*	*2.47*
22-A	5	1.6	HALAR																	*1/3*	*1.57*
23-B	5	2	KAPTON											*1/3*	*1.59*					*2/3*	*2.83*
24-C	5	2.4	METAL																		
NUM. TOT.				5		5		*7*		11		8		*9*		14		18		*20*	
	**D.** **EX.**	**d.**	**MATERIAL**	**SINE 18 s**		**SQUARE 18 s (90 s)—5th**		***MFS 18 s*** ***(45–90 s)*** **5th**		**SINE 30 s**		**SQUARE** **30 s—3rd**		***MFS 30 s*** ***(45–90 s)*** **—3rd**		**SINE 90 s**		**SQUARE 90 s**		***MFS 90 s*** ***(45–90 s)***	
				**P**	**CNR (m. v.)**	**P**	**CNR** **(m. v.)**	***P***	***CNR*** ***(m. v.)***	**P**	**CNR** **(m. v.)**	**P**	**CNR** **(m. v.)**	***P***	***CNR*** ***(m. v.)***	**P**	**CNR** **(m. v.)**	**P**	**CNR** **(m. v.)**	***P***	***CNR*** ***(m. v.)***
1-A	17	0.4	HALAR													1	−6.44	1	−5.07	*1*	*−4.51*
2-B	17	0.8	KAPTON	1	9.26	1	4.86	*2/3*	*3.44*	1	7.76	1	5.18	*1*	*4.29*	1	−3.27	2/3	−2.90	*2/3*	*−3.84*
3-C	17	1.2	METAL	1	6.10	1	3.10	*2/3*	*2.04*	1	8.63	1	4.98	*1*	*3.74*	1	3.28	1	3.36	*1/3*	*1.30*
4-A	17	1.6	HALAR	2/3	1.90					1	3.44	2/3	2.18	*1/3*	*1.81*	1/3	1.78				
5-B	17	2	KAPTON							1	3.50	1/3	1.75	*1/3*	*1.09*	1	3.73	1	3.16		
6-C	17	2.4	METAL							1/3	1.75					1	3.49	1	2.74		
7-A	12	0.4	HALAR	2/3	2.02	1/3	1.33	*1/3*	*−0.53*					*1/3*	*−1.23*	1	−8.25	1	−9.37	*1*	*−7.30*
8-B	12	0.8	KAPTON	1	7.10	1	4.01	*1*	*2.90*	1	5.69	1	3.65	*2/3*	*3.15*	1	−3.51	1	−4.54	*1*	*−4.22*
9-C	12	1.2	METAL	1	4.73	1	3.04	*2/3*	*2.24*	1	6.01	1	4.24	*1*	*4.62*						
10-A	12	1.6	HALAR	1/3	1.80					1	2.57	1/3	1.28	*2/3*	*2.38*						
11-B	12	2	KAPTON							1/3	1.77			*1/3*	*1.43*	1/3	1.76	1/3	1.82	*1/3*	*1.64*
12-C	12	2.4	METAL													2/3	2.39	1/3	1.49	*1*	*2.55*
13-A	9	0.4	HALAR							1	−2.51	1	−2.36			1	−9.09	1	−8.62	*1*	*−7.76*
14-B	9	0.8	KAPTON	1	5.27	1	3.63	*2/3*	*2.51*	1	3.33	1	2.55	*1/3*	*1.96*	1	−3.30	1	−3.84	*1*	*−2.77*
15-C	9	1.2	METAL	1	3.10	1	2.30	*1/3*	*1.67*	1	3.68	1	2.97	*2/3*	*2.50*						
16-A	9	1.6	HALAR							2/3	2.29			*1/3*	*1.67*					*1/3*	*1.59*
17-B	9	2	KAPTON							1/3	1.90			*1/3*	*1.08*	1/3	2.04	1/3	1.89	*2/3*	*2.23*
18-C	9	2.4	METAL													2/3	2.43	2/3	2.25	*2/3*	*2.41*
19-A	5	0.4	HALAR					*1/3*	*−1.23*	2/3	−2.11			*1/3*	*−1.16*	1	−5.89	1	−5.57	*1*	*−4.11*
20-B	5	0.8	KAPTON	2/3	1.54	1/3	1.73					1/3	1.66								
21-C	5	1.2	METAL							1/3	1.86	2/3	2.23								
22-A	5	1.6	HALAR																		
23-B	5	2	KAPTON			1/3	0.74			1/3	1.34										
24-C	5	2.4	METAL																		
NUM. TOT.				10		9		*8*		17		12		*14*		15		14		*13*	

## Data Availability

Data is contained within the article.
